# Outcomes of Single Blastocyst Transfer Versus Double Cleavage-Stage Embryo Transfer in Young Women with Recurrent Implantation Failure: A Secondary Analysis of a Multicenter Randomized Trial

**DOI:** 10.3390/jcm15145544

**Published:** 2026-07-15

**Authors:** Yuan Wang, Changyang Xu, Yaqiong He, Jiaan Huang, Yun Sun, Yao Lu, Zhe Wei

**Affiliations:** Department of Reproductive Medicine, Shanghai Key Laboratory for Assisted Reproduction and Reproductive Genetics, Ren Ji Hospital, Shanghai Jiao Tong University School of Medicine, Shanghai 200135, China; sooxiaoyuanzi@163.com (Y.W.); xcy_929@outlook.com (C.X.); heya.qiong@163.com (Y.H.); huangjiaan168@163.com (J.H.); syun163@163.com (Y.S.)

**Keywords:** recurrent implantation failure, in vitro fertilization, blastocyst transfer, cleavage-stage embryo transfer, live birth

## Abstract

**Objectives:** Although blastocyst transfer may improve embryo selection and early pregnancy outcomes, its impact on live birth in women with recurrent implantation failure (RIF) remains uncertain. This study compared reproductive and perinatal outcomes between single blastocyst transfer (SBT) and double cleavage-stage embryo transfer (DET) among women younger than 38 years with RIF. **Methods:** Secondary observational analysis of a multicenter, randomized, double-blind, placebo-controlled clinical trial conducted across eight academic fertility centers in China. In the original trial, patients with RIF under 38 years of age were enrolled and randomized to receive either prednisone or placebo. For the present analysis, a total of 582 patients were categorized according to the embryo transfer strategy. The SBT-versus-DET comparison was not randomized, and the two strategies differed in both embryo developmental stage and number of embryos transferred. **Results:** No statistically significant difference in live birth rate was observed between the SBT and DET groups (41.8% vs. 38.4%; *p* = 0.471), despite a higher biochemical pregnancy rate in the SBT group (59.9% vs. 45.7%, *p* = 0.003). Pregnancy loss was more frequent in the SBT group than in the DET group (30.2% vs. 15.9%; *p* = 0.018), particularly biochemical pregnancy loss (15.5% vs. 5.8%; *p* = 0.036), whereas twin live birth was less frequent (0.7% vs. 9.9%; *p* < 0.001). After adjustment, no significant difference in live birth rate was observed between DET and SBT (adjusted OR 1.04, 95% CI 0.68–1.57). In addition, Day 5 SBT showed a higher live birth rate than Day 6 SBT, whereas pregnancy loss rates were similar between Day 5 and Day 6 SBT cycles. Other exploratory sensitivity and stratified analyses showed broadly consistent findings. **Conclusions:** Although SBT is generally preferred in clinical practice, our study found no statistically significant difference in live birth rates between SBT and DET among women younger than 38 years with RIF. Given that pregnancy loss was observed more frequently in the SBT group, whereas twin live births were less frequent, our findings support individualized counseling rather than uniform use of either transfer strategy. However, given the non-randomized nature of this comparison, these findings should be interpreted with caution.

## 1. Introduction

In vitro fertilization–embryo transfer (IVF-ET) is an established treatment for infertility and has been increasingly used worldwide [1,2,3]. Despite advances in assisted reproductive technologies, 50% to 60% of IVF cycles still fail to achieve implantation [4,5]. Recurrent implantation failure (RIF), defined as the absence of clinical pregnancy following multiple embryo transfers, continues to pose significant challenges for both patients and clinicians [6]. Although various therapeutic interventions have been proposed and applied, only a few are supported by high-quality evidence [7].

Embryo transfer stage is an important determinant of IVF outcomes. Cleavage-stage transfer was historically preferred because extended culture may increase embryo attrition and reduce the opportunity of transfer. With advances in culture systems, however, blastocyst transfer has become increasingly common [8,9]. Culturing embryos to the blastocyst stage is thought to facilitate the selection of embryos with greater developmental potential [10,11]. In addition, blastocyst transfer may better synchronize embryo transfer with the physiological timing of uterine entry, which usually occurs around day 5 after fertilization [12]. These potential advantages have contributed to the increasing use of blastocyst transfer in clinical practice [11].

However, in women with RIF, whether embryos should be transferred at the cleavage stage or the blastocyst stage remains controversial. Two observational cohort studies reported that blastocyst transfer was associated with higher rates of implantation and clinical pregnancy [13,14]. In contrast, a prospective randomized trial involving 54 patients demonstrated similar live birth rates (LBRs) following fresh blastocyst and cleavage-stage embryo transfers [15]. Despite their compelling nature, each study has limitations, including a small sample size and incomplete reporting of prenatal outcomes and LBR. The current evidence therefore remains insufficient. Although some clinical guidelines and expert consensus statements indicate that blastocyst transfer may improve pregnancy outcomes in patients with RIF, they do not specifically recommend an embryo transfer strategy for young RIF patients [6,7]. Furthermore, prolonged in vitro embryo culture may also be associated with potential risks [16,17], making blastocyst culture a double-edged sword.

Recently, our team conducted a large-scale, multicenter, prospective, randomized controlled trial to determine whether oral prednisone could improve the live birth rate in patients with RIF [18]. In the original trial, participants were randomly assigned in a double-blind manner to receive either prednisone or a matching placebo. The findings indicated that prednisone did not improve the live birth rate in patients with RIF but increased the risks of biochemical pregnancy loss and preterm birth. The present study is a secondary analysis of that randomized controlled trial (RCT), aiming to compare the pregnancy outcomes, perinatal and neonatal complications between single blastocyst transfer (SBT) and double cleavage-stage embryo transfer (DET) in RIF patients younger than 38 years old. Our findings may provide exploratory evidence to help optimize embryo transfer strategies for young women with RIF.

## 2. Materials and Methods

### 2.1. Study Subjects

This research represents a secondary observational analysis of a multicenter, randomized, double-blind, placebo-controlled clinical trial involving RIF patients, conducted across eight academic fertility centers in China (registration number: ChiCTR1800018783) [18].

The design, rationale, and outcomes of the original trial have been detailed in prior publications [18,19]. Briefly, eligible participants were women under 38 years old at the time of oocyte retrieval who planned to undergo another cycle of frozen-thawed embryo transfer (FET). They were required to have at least one blastocyst or two cleavage-stage embryos of good quality following IVF, or intracytoplasmic sperm injection (ICSI). In our study, RIF was defined as at least two previous unsuccessful embryo transfer attempts, with a cumulative total of at least three high-quality embryos transferred. These previous failed transfers could have involved cleavage-stage embryos, blastocysts, or both. High-quality embryos were defined as blastocysts graded ≥4BC by the Gardner scoring system [20], and cleavage-stage embryos with 7–10 cells graded 3 or 4, or I or II [21]. Women were excluded if they had a history of recurrent pregnancy loss (defined as two or more failed clinical pregnancies), a thin endometrium (less than 6 mm), abnormal parental karyotyping, contraindications to assisted reproductive technology (ART) or pregnancy, or any diagnosed conditions that affect the uterine cavity.

In the original trial, participants were randomly assigned in a 1:1 ratio to receive either 10 mg prednisone or matching placebo once daily. Study medication was initiated on the day of endometrial preparation for FET and continued after embryo transfer; if pregnancy was confirmed, medication was continued until 12 weeks of gestation. Randomization was performed using a computer-generated sequence with permuted blocks, and was stratified by embryo stage and study center.

**Figure 1:**
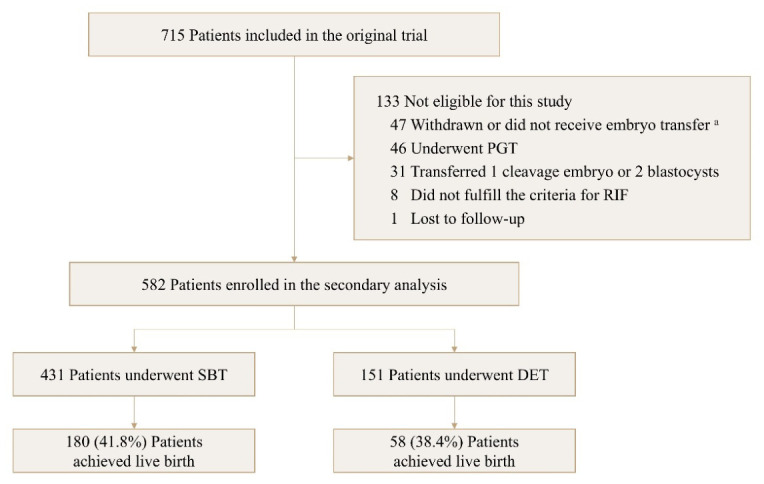
Patient Flow of the study. A total of 582 out of 715 RIF patients were included in the present analysis, with specific exclusion criteria. ^a^ Transfers canceled because of thin endometrium, elevated progesterone level, personal issues, or other adverse effects. Abbreviations: PGT, preimplantation genetic testing; RIF, recurrent implantation failure; DET, double cleavage-stage embryo transfer; SBT, single blastocyst-stage embryo transfer.

This secondary analysis only included patients who underwent IVF or ICSI and underwent transfer of either one blastocyst or two cleavage-stage embryos. Patients were categorized into two groups according to the developmental stage of embryos transferred: the SBT group (*n* = 431) and the DET group (*n* = 151). Patients were excluded if they did not undergo embryo transfer, underwent preimplantation genetic testing (PGT), transferred one cleavage-stage embryo or two blastocysts, did not meet the RIF diagnostic criteria, or were lost to follow-up (Figure 1). This study was reported in accordance with the Transparent Reporting of Evaluations with Nonrandomized Designs (TREND) statement. The completed TREND checklist is provided as Supplementary File S1.

### 2.2. ART Procedures

Each patient received a personalized ART protocol, which included ovarian stimulation and fertilization procedures. Decisions regarding these procedures, whether to proceed with IVF or ICSI, were made based on a comprehensive evaluation of the patient’s medical history and semen analysis results on the day of oocyte retrieval. The embryos were cultured in vitro according to the clinical routine of each participating reproductive center. As previously described, all embryos transferred in this study met predefined good-quality criteria. The choice between SBT and DET was primarily determined by the highest-quality embryos available to each participant at enrollment.

### 2.3. Frozen–Thawed Embryo Transfer

All participants underwent FET using a standardized endometrial preparation regimen. Initially, 2 to 8 mg of estradiol valerate (Progynova, Delpharm Lille SAS, Leverkusen, Germany) and/or estradiol tablets (Femoston, Abbott, Lake County, IL, USA) were administered starting from days 2 to 5 of the menstrual cycle or after suppression with a long-acting gonadotropin-releasing hormone agonist (GnRH-a) (Decapeptyl, Ferring Pharmaceuticals, Saint-Prex, Switzerland). Upon reaching an adequate endometrial thickness, oral dydrogesterone (Duphaston, Abbott, Lake County, IL, USA) at 10 mg twice daily and vaginal progesterone gel (Crinone, Merck Serono, Feltham, UK) at 90 mg daily were introduced for luteal phase support. For the DET group, two high-quality cleavage-stage embryos were transferred after 3 days of progesterone administration, while in the SBT group, a single high-quality blastocyst was transferred after 5 days of progesterone administration. Oral estradiol was gradually tapered off, and luteal phase support was continued until 8 to 12 weeks of gestation if clinical pregnancy was confirmed. Pregnancy and neonatal outcomes were derived from obstetric and neonatal medical records, with all pregnancies monitored to term.

### 2.4. Measured Outcomes

The primary outcome was the live birth rate per embryo transfer, defined as the number of neonates born alive at 28 weeks of gestation or later. Secondary outcomes encompassed biochemical pregnancy, identified by a serum β-hCG level of ≥10 mIU/mL measured 12–15 days post-embryo transfer, and clinical pregnancy, confirmed by the detection of a gestational sac via transvaginal ultrasound approximately 35 days after embryo transfer. Pregnancy loss included any pregnancies that resulted in spontaneous or therapeutic abortion at any stage of gestation. Biochemical pregnancy loss was defined as a biochemical pregnancy that did not progress to clinical pregnancy. Clinical pregnancy loss was defined as a clinical pregnancy that did not result in live birth. Additionally, perinatal outcomes and fetal adverse events were tracked as part of the secondary outcomes.

### 2.5. Statistical Methods

Data were analyzed utilizing SPSS software (SPSS Inc., version 21.0; Chicago, IL, USA). Continuous variables were expressed as mean ± standard deviation (SD) and analyzed using a Student’s *t*-test for normally distributed data, and as median and interquartile range (IQR) using the Wilcoxon rank-sum test for data that was not normally distributed. Categorical variables were reported as frequencies and percentages and analyzed using either Fisher’s exact test or the Pearson χ^2^ test to evaluate between-group differences. Multivariate logistic regression was applied to control for potential confounders including age at oocyte retrieval, previous live births, duration of infertility, basal follicle-stimulating hormone (FSH) levels, number of previous oocyte retrievals, number of previous failed embryo transfer cycles, study center and randomized prednisone/placebo allocation in the original trial. To address the potential influence of prednisone allocation, an exploratory placebo-only sensitivity analysis was performed. To further minimize bias due to baseline imbalance, propensity score matching (PSM) was conducted using a 1:1 nearest-neighbor algorithm. Propensity scores were estimated using logistic regression based on age at oocyte retrieval, previous live birth, duration of infertility, basal FSH, previous oocyte retrievals, previous failed embryo transfer cycles, and randomized prednisone/placebo allocation in the original trial. Post-matching covariate balance was assessed using standardized mean differences (SMDs), with an SMD < 0.2 considered acceptable for covariate balance [22]. Baseline characteristics were further compared after matching to confirm the balance of all major confounders. Additional analyses were performed according to blastocyst developmental day. Furthermore, stratified analyses were conducted by age at oocyte retrieval (<35 or ≥35), number of previous oocyte retrievals (1 or ≥2), number of previous failed embryo transfer cycles (2 or ≥3). A *p*-value of less than 0.05 was considered statistically significant.

## 3. Results

### 3.1. Baseline Characteristics

A total of 582 of the 715 RIF patients were included in the present analysis, with the exclusion criteria detailed in Figure 1. Among the included patients, 431 (74.1%) underwent single blastocyst transfer, while 151 (25.9%) underwent double cleavage-stage embryo transfer.

Comparison of baseline data between the two groups (Table 1) showed that patients in the SBT group were younger, had more previous live births, shorter durations of infertility, lower baseline levels of FSH, and fewer previous oocyte retrievals, but had experienced more previous embryo transfer failures. These findings indicate that the two groups differed in prognostic characteristics. Other clinical baseline characteristics including BMI, history of previous abortion, indications for IVF, and the number of previous embryos transferred were similar between the two groups. The proportion of participants randomized to prednisone in the original trial was similar between the SBT and DET groups (48.0% vs. 49.7%, *p* = 0.728).

### 3.2. Embryo Transfer Cycle Characteristics and Clinical Outcomes

As shown in Table 2, endometrial thickness was similar between the two groups. All patients in the DET group underwent Day-3 cleavage-stage embryos transfer. In the SBT group, 68.2% of patients underwent Day-5 blastocyst transfer, whereas 31.8% underwent Day-6 blastocyst transfer.

As shown in Table 3, no statistically significant difference in live birth rate was observed between SBT and DET groups (41.8% vs. 38.4%; *p* = 0.471). Clinical pregnancy rates were also similar between groups (50.6% vs. 43.0%; *p* = 0.111). The SBT group had a higher biochemical pregnancy rate than the DET group (59.9% vs. 45.7%, *p* = 0.003). The pregnancy loss rate was higher in the SBT group than in the DET group, whether calculated per pregnancy (30.2% vs. 15.9%; *p* = 0.018) or per embryo transfer (18.1% vs. 7.3%; *p* = 0.002), particularly biochemical pregnancy loss rate (per biochemical pregnancy: 15.5% vs. 5.8%; *p* = 0.036 or per embryo transfer: 9.3% vs. 2.6%; *p* = 0.007). The twin live birth rate was lower in the SBT group than in the DET group (0.7% vs. 9.9%; *p* < 0.001).

Multivariate logistic regression was performed to minimize confounding factors by adjusting for age at oocyte retrieval, previous live birth, duration of infertility, baseline FSH level, number of previous oocyte retrievals, previous failed embryo transfer cycles, study center and prednisone/placebo allocation in the original trial. In the adjusted model (Table 4), DET was associated with a similar live birth rate compared with SBT (adjusted OR 1.04, 95% CI 0.68–1.57). DET was also associated with similar odds of clinical pregnancy (adjusted OR 0.83, 95% CI 0.56–1.25), but lower odds of pregnancy loss (adjusted OR 0.41, 95% CI 0.19–0.86).

In an exploratory propensity score–matched sensitivity analysis, 151 patients in the SBT group were matched to 151 patients in the DET group. After matching, measured baseline characteristics were better balanced between groups. No statistically significant difference in live birth rate was observed between SBT and DET after matching (42.4% vs. 38.4%, *p* = 0.482). The SBT group continued to show a higher biochemical pregnancy rate (60.3% vs. 45.7%, *p* = 0.011) and pregnancy loss rate (29.7% vs. 15.9%, *p* = 0.043), whereas clinical pregnancy rate was not significantly different (51.7% vs. 43.0%, *p* = 0.134) (Supplementary Table S1).

In an exploratory placebo-only sensitivity analysis restricted to participants randomized to placebo in the original trial (Supplementary Table S2), no statistically significant difference in live birth rate was observed between the SBT and DET groups (42.9% vs. 35.5%, *p* = 0.262). Pregnancy loss rates were higher in the SBT group than in the DET group (26.7% vs. 6.9%, *p* = 0.022), together with a higher biochemical pregnancy rate (58.5% vs. 38.2%, *p* = 0.002).

Follow-up of perinatal and neonatal complications showed that the SBT group had decreased rates of preterm birth and low birth weight in newborns. However, when singletons and twins were analyzed separately, no differences were observed in the rates of preterm birth or newborn birth weight between the two groups. Furthermore, the incidences of gestational diabetes, premature rupture of membranes, gestational hypertension, preeclampsia, and congenital anomalies in newborns were similar between the two groups (Supplementary Table S3).

### 3.3. Exploratory Analyses by Blastocyst Developmental Day

We further performed exploratory analyses according to blastocyst developmental day. Pregnancy outcomes were compared among Day 5 SBT, Day 6 SBT, and DET cycles (Supplementary Table S4). The biochemical pregnancy rates were 66.0%, 46.7%, and 45.7% in the Day 5 SBT, Day 6 SBT, and DET groups, respectively (*p* < 0.001). The corresponding clinical pregnancy rates were 55.8%, 39.4%, and 43.0%, respectively (*p* = 0.022). The live birth rates were 46.3%, 32.1%, and 38.4%, respectively (*p* = 0.016). Pregnancy loss rates were numerically higher in both Day 5 SBT and Day 6 SBT cycles than in DET cycles (29.9%, 31.3%, and 15.9%, respectively; *p* = 0.059).

In adjusted pairwise analyses (Supplementary Table S5), Day 5 SBT was associated with higher odds of biochemical pregnancy (adjusted OR 2.39, 95% CI 1.56–3.68), clinical pregnancy (adjusted OR 2.02, 95% CI 1.32–3.11), and live birth (adjusted OR 1.91, 95% CI 1.22–3.00) compared with Day 6 SBT. However, pregnancy loss was comparable between Day 5 and Day 6 SBT cycles (adjusted OR 0.85, 95% CI 0.44–1.63). Compared with Day 5 SBT, DET was associated with lower odds of biochemical pregnancy, clinical pregnancy, and pregnancy loss, but similar odds of live birth. Compared with Day 6 SBT, DET showed similar odds of biochemical pregnancy, clinical pregnancy, and live birth, but lower odds of pregnancy loss. These exploratory findings were consistent with the possibility that Day 6 blastocyst transfer may partly explain the lower live birth rate within the SBT group, whereas the higher pregnancy loss observed in the overall SBT group did not appear to be solely attributable to Day 6 blastocyst transfers. However, because the analysis was not randomized and was limited by subgroup sample size, these results should be considered hypothesis-generating.

### 3.4. Stratified Analysis

**Figure 2:**
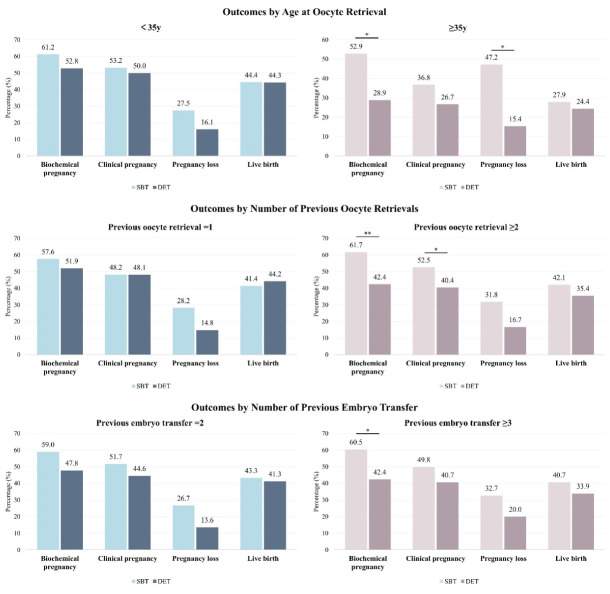
Stratified analysis. Stratified analyses were conducted based on age at oocyte retrieval, number of previous oocyte retrievals, and number of previous embryo transfers. * *p* < 0.05, ** *p* < 0.01. Abbreviations: SBT, single blastocyst-stage embryo transfer; DET, double cleavage-stage embryo transfer.

Stratified analyses were conducted according to age at oocyte retrieval, number of previous oocyte retrievals, and number of previous embryo transfers, as shown in Figure 2 and Supplementary Table S6. Among women under the age of 35, as well as those with only one previous oocyte retrieval or two previous embryo transfers, the rates of biochemical pregnancy, clinical pregnancy, and live birth rate were all comparable between the two groups. In contrast, among women aged 35 years or older, those with two or more previous oocyte retrievals, or those with three or more previous embryo transfers, biochemical pregnancy rate was significantly higher in the SBT group than in the DET group. However, this did not result in a difference in live birth rate. Moreover, a significantly increased risk of pregnancy loss was observed in the SBT group among women aged 35 years or older, while among other strata, a similar but statistically non-significant trend was noted. No statistically significant difference in live birth rate was observed across strata; however, these subgroup findings should be considered exploratory because of limited sample size and event numbers.

## 4. Discussion

To the best of our knowledge, this is the first study to compare pregnancy outcomes between single blastocyst and double cleavage-stage embryo transfer in FET cycles for young women with RIF. In this secondary observational analysis of a multicenter randomized trial cohort [18], no statistically significant difference in live birth rate was observed between SBT and DET groups among women younger than 38 years with RIF undergoing FET. Pregnancy loss was more frequently observed in the SBT group, whereas twin live birth was less frequent. Because the original trial showed that prednisone did not improve live birth but was associated with increased risks of biochemical pregnancy loss and preterm delivery, we considered the randomized prednisone/placebo allocation to be clinically relevant in the present analysis. Importantly, prednisone allocation was balanced between the SBT and DET groups. After further adjusting for randomized treatment allocation in the original trial, the main findings remained materially unchanged.

Few studies have evaluated pregnancy outcomes related to cleavage and blastocyst-stage embryo transfer among the RIF population. F. Guerif et al. [13] included 276 IVF patients with at least two failed embryo transfer cycles in a prospective non-randomized study. The authors reported higher clinical pregnancy rate, implantation rate, and live birth rate after blastocyst transfer, although their study only investigated day 2 cleavage-stage embryo transfer. In a small randomized trial, Eliahu Levitas et al. [15] reported that while the blastocyst transfer group had a greater implantation rate, neither the clinical pregnancy rate nor the live birth rate improved. However, both studies focused on fresh embryo transfers, which differs from our study, and the potential interference of endometrial receptivity in fresh cycles cannot be ruled out. Recently, Zhang X et al. [14] reported that in the first FET cycle for RIF patients, SBT resulted in higher clinical pregnancy rate and ongoing pregnancy rate than DET, although there was insufficient information on the live birth rate. Additionally, the study also included women who were older than 40. It is well-known that the incidence of aneuploidy in embryos increases with maternal age [23,24], and blastocyst culture can help select against some aneuploid embryos that are less likely to develop to the blastocyst stage [25], thereby improving the selection of embryos for transfer and benefiting pregnancy outcomes. However, our study population consists of women younger than 38 years old, in whom embryonic aneuploidy is less common, potentially undermining the benefits of blastocyst culture. Cornelisse S et al. [26] conducted a large-scale RCT in the general population of IVF and reported similar findings to ours. The cleavage-stage embryo transfer group, compared to the blastocyst transfer group, had a lower live birth rate for fresh embryo transfer but underwent more embryo transfers and transferred more embryos. When considering the cumulative live birth rates across a single oocyte retrieval cycle, there were no differences between the groups, indicating that increasing the number of embryos transferred can achieve identical pregnancy outcomes to blastocyst transfer.

It is not surprising that the biochemical pregnancy rate in the SBT group is significantly elevated, as observed in the general IVF population. However, in our RIF cohort, the higher biochemical pregnancy rate in the SBT group did not translate into a higher live birth rate. Instead, pregnancy loss was more frequently observed in the SBT group, particularly biochemical pregnancy loss. Similar results have been reported by another study [27], which found a higher early miscarriage rate in double blastocyst transfer group compared to double cleavage-stage embryo transfer group (17.2% vs. 8.1%). This finding should not be interpreted as evidence that blastocyst transfer itself causes miscarriage. Several alternative explanations should be considered. First, the SBT group was heterogeneous in terms of blastocyst developmental day. Day 5 SBT showed higher pregnancy rate and live birth rate than Day 6 SBT, suggesting greater developmental competence; however, pregnancy loss rates were similar between Day 5 and Day 6 SBT cycles, indicating that the higher pregnancy loss observed in the overall SBT group was not solely driven by Day 6 blastocyst transfer. Second, RIF itself is biologically heterogeneous and may involve endometrial receptivity, embryo–endometrium synchrony, or uterine factors. Therefore, the pregnancy-loss pattern observed in this study likely reflects the interaction of multiple factors rather than a direct detrimental effect of blastocyst transfer.

We also evaluated perinatal and neonatal complications. The crude rates of preterm birth and low birth weight were higher in the DET group than in the SBT group, which was likely attributable to the higher twin live birth rate in the DET group [28]. After stratification by singleton and twin live births, no clear between-group differences in preterm birth or birthweight were observed. Some studies suggest that extended embryo culture to the blastocyst stage, which prolongs in vitro culture time, may affect the development of trophoblasts via epigenetic mechanisms [29], potentially leading to placenta-derived diseases [30]. Although six cases of pre-eclampsia occurred in the SBT group and none in the DET group, the small number of events precludes firm conclusions. Furthermore, no differences were observed in other placenta-related diseases such as gestational hypertension between the two groups.

However, this study also has some limitations. Firstly, the original trial was not designed to compare the pregnancy outcomes between SBT and DET. Consequently, embryo transfer strategy was not randomized, and selection bias cannot be ignored in this secondary observational analysis. Patients in the DET group were generally older, had higher baseline FSH levels, longer durations of infertility, more previous oocyte retrievals and fewer embryo transfers. This is consistent with routine clinical practices, in which clinicians typically freeze more high-quality embryos on day 3 post-fertilization for patients with poorer prognoses [31,32,33,34], providing more opportunities for embryo transfer and thereby increasing the likelihood of successful pregnancy by transferring more embryos. Although multivariable regression and propensity score matching were used to account for measured baseline imbalances, these approaches cannot eliminate residual confounding from unmeasured factors such as detailed embryo grading, embryo availability, clinician judgment, and center-specific practice. These factors may have influenced transfer strategy selection and could not be completely adjusted. Secondly, the stratified analysis revealed that certain subgroups had smaller sample sizes. This could result in insufficient statistical power to reach clear conclusions. These findings should therefore be considered exploratory and hypothesis-generating. Interpretation of perinatal and neonatal outcomes was limited by the relatively small number of live birth cycles. Therefore, comparisons stratified by singleton and twin live births should be interpreted cautiously. Thirdly, this study evaluated the live birth rate per embryo transfer rather than cumulative live birth rate per oocyte retrieval, as the original trial did not capture data on cumulative live birth rate. This distinction is important when comparing cleavage-stage and blastocyst-stage transfer strategies, because embryo attrition during extended culture may influence the number of embryos available for transfer and the cumulative probability of live birth from a single retrieval. Furthermore, as RIF definitions differ substantially across existing studies, our results may not be reliably extrapolated to other populations. Finally, this study could not isolate the independent effects of embryo developmental stage and embryo number. Therefore, the observed findings should not be interpreted as evidence for the independent effect of blastocyst-stage transfer itself and the study should be considered exploratory. Given that the relatively wide confidence intervals observed in our analyses cannot completely rule out a clinically meaningful difference in live birth rates between groups, a future definitive randomized trial is warranted to verify our results. The trial should enroll RIF patients who are eligible for both SBT and DET, use a prespecified and standardized RIF definition, and stratify randomization by key prognostic factors such as age, study center, and the number of previous failed embryo transfers. In addition, future trials should evaluate cumulative live birth per oocyte retrieval, healthy singleton live birth, pregnancy loss, and perinatal and neonatal outcomes.

## 5. Conclusions

In women younger than 38 years with recurrent implantation failure, no statistically significant difference in live birth rate was observed between SBT and DET. Pregnancy loss was observed more frequently in the SBT group, whereas twin live birth was less frequent. Clinically, these findings support individualized counseling rather than uniform use of either transfer strategy. Pending definitive randomized evidence, decisions should incorporate patient prognosis, embryo availability and quality, blastocyst developmental day, and prior transfer history.

## Figures and Tables

**Table 1:** Baseline Characteristics.

	SBT (*n* = 431)	DET (*n* = 151)	*p*-Value
Baseline Characteristics			
Female age, years			
At consent	32 (30–34)	33 (30–36)	0.035
At oocyte retrieval	31 (29–34)	32 (29–35)	0.043
BMI, kg/m^2^	22.7 ± 3.1	22.5 ± 3.1	0.545
Previous live birth	68 (15.8)	13 (8.6)	0.029
Previous abortion	126 (29.2)	51 (33.8)	0.297
Duration of infertility, years	4 (2–6)	5 (3–7)	0.011
Indications for IVF			0.974
Tubal factor	172 (39.9)	62 (41.1)	
Male factor	63 (14.6)	21 (13.9)	
Ovulatory dysfunction	34 (7.9)	12 (7.9)	
Endometriosis	11 (2.6)	2 (1.3)	
Combined factors	134 (31.1)	48 (31.8)	
Unexplained	17 (3.9)	6 (4.0)	
Basal follicle-stimulating hormone, mIU/mL	6.5 ± 2.0	7.0 ± 2.4	0.026
Previous oocyte retrievals	2 (1–2)	2 (1–3)	0.005
Previous failed embryo transfer cycles	3 (2–3)	2 (2–3)	<0.001
Previous embryos transferred	4 (3–5)	4 (3–5)	0.249
Previous good-quality embryos transferred	4 (3–5)	4 (3–4)	0.515
Fertilization method			0.190
IVF	288 (66.8)	92 (60.9)	
ICSI	143 (33.2)	59 (39.1)	
Original-trial prednisone allocation, No. (%)	207 (48.0)	75 (49.7)	0.728

Abbreviations: SBT, single blastocyst-stage embryo transfer; DET, double cleavage-stage embryo transfer; BMI, body mass index; No, number; IVF, in vitro fertilization; ICSI, intracytoplasmic sperm injection; No, number. Prednisone allocation refers to randomized treatment assignment in the original placebo-controlled trial.

**Table 2:** Frozen–thawed embryo transfer.

	SBT (*n* = 431)	DET (*n* = 151)	*p*-Value
Endometrial thickness, mm	9.0 ± 1.5	9.2 ± 1.7	0.167
Stage of embryo (s) transferred, No. (%)			<0.001
D3		151 (100.0)	
D5	294 (68.2)		
D6	137 (31.8)		

Abbreviations: SBT, single blastocyst-stage embryo transfer; DET, double cleavage-stage embryo transfer; No, number.

**Table 3:** Pregnancy Outcomes.

	SBT (*n* = 431)	DET (*n* = 151)	RR (95% CI)	*p*-Value
Biochemical pregnancy	258 (59.9)	69 (45.7)	0.76 (0.63–0.92)	0.003
Clinical pregnancy	218 (50.6)	65 (43.0)	0.85 (0.69–1.05)	0.111
Pregnancy loss				
Per biochemical pregnancy	78/258 (30.2)	11/69 (15.9)	0.53 (0.30–0.93)	0.018
Per embryo transfer	78 (18.1)	11 (7.3)	0.40 (0.22–0.74)	0.002
Biochemical pregnancy loss				
Per biochemical pregnancy	40/258 (15.5)	4/69 (5.8)	0.37 (0.14–1.01)	0.036
Per embryo transfer	40 (9.3)	4 (2.6)	0.29 (0.10–0.78)	0.007
Clinical pregnancy loss				
Per clinical pregnancy	38/218 (17.4)	7/65 (10.8)	0.62 (0.29–1.32)	0.197
Per embryo transfer	38 (8.8)	7 (4.6)	0.53 (0.24–1.15)	0.098
Live birth	180 (41.8)	58 (38.4)	0.92 (0.73–1.16)	0.471
Singleton	177 (41.1)	43 (28.5)	0.69 (0.53–0.91)	0.006
Twin	3 (0.7)	15 (9.9)	14.27 (4.19–48.61)	<0.001

Abbreviations: SBT, single blastocyst-stage embryo transfer; DET, double cleavage-stage embryo transfer; RR, risk ratio. Risk ratios were calculated with the SBT group as the reference.

**Table 4:** Univariate and Multivariate Analysis of Pregnancy Outcomes.

Outcome	Crude OR (95% CI)	*p*-Value	Adjusted OR (95% CI)	*p*-Value
Biochemical pregnancy	0.56 (0.39 to 0.82)	0.003	0.67 (0.45 to 1.00)	0.050
Clinical pregnancy	0.74 (0.51 to 1.07)	0.112	0.83 (0.56 to 1.25)	0.378
Pregnancy loss	0.44 (0.22 to 0.88)	0.020	0.41 (0.19 to 0.86)	0.018
Biochemical pregnancy loss	0.34 (0.12 to 0.97)	0.044	0.38 (0.12 to 1.15)	0.086
Clinical pregnancy loss	0.57 (0.24 to 1.35)	0.202	0.44 (0.17 to 1.10)	0.079
Live birth	0.87 (0.60 to 1.27)	0.471	1.04 (0.68 to 1.57)	0.867

Abbreviations: OR, odds ratio; CI, confidence interval. Data were adjusted for age at oocyte retrieval, previous live birth, duration of infertility, basal follicle-stimulating hormone, previous oocyte retrievals, previous failed embryo transfer cycles, study center and randomized prednisone/placebo allocation in the original trial. Single blastocyst transfer cycles were used as a reference.

## Data Availability

The data that support the findings of this study are available from the corresponding author upon reasonable request.

## Supplementary Materials

The following supporting information can be downloaded at: https://www.mdpi.com/article/10.3390/jcm15145544/s1, Table S1: Pregnancy outcomes before and after propensity score matching; Table S2: Pregnancy outcomes between SBT and DET among participants randomized to placebo in the original trial; Table S3: Perinatal Outcomes; Table S4: Pregnancy outcomes among Day 5 SBT, Day 6 SBT, and DET cycles; Table S5: Univariate and Multivariate Analysis with regard to Pregnancy Outcomes among Day 5 SBT, Day 6 SBT, and DET cycles; Table S6: Stratified analysis. File S1: TREND Statement Checklist. Reference [35] is cited in Supplementary Materials.

## Abbreviations

The following abbreviations are used in this manuscript: RIF, recurrent implantation failure; DET, double cleavage-stage embryo transfer; SBT, single blastocyst-stage embryo transfer; IVF-ET, in vitro fertilization–embryo transfer; LBRs, live birth rates; RCT, randomized controlled trial; ICSI, intracytoplasmic sperm injection; ART, assisted reproductive technology; PGT, preimplantation genetic testing; GnRH-a, gonadotropin-releasing hormone agonist; FSH, follicle-stimulating hormone. PSM, propensity score matching; SMDs, standardized mean differences.
