# Supplementary material for: Outcomes of Single Blastocyst Transfer Versus Double Cleavage-Stage Embryo Transfer in Young Women with Recurrent Implantation Failure: A Secondary Analysis of a Multicenter Randomized Trial
Source: J Clin Med. 2026 Jul 15;15(14):5544. doi: 10.3390/jcm15145544 (PMC13413220; doi:10.3390/jcm15145544)
Supplement: Supplementary file 1 [file jcm-15-05544-s001.zip › jcm-4390551-supplementary.pdf]

Supplementary Table S1. Pregnancy outcomes before and after propensity score matching

	SBT unmatched (n=431)	DET (n=151)	*P*-value Unmatched	SBT matched (n=151)	*P*-value Matched
**Baseline characteristics**					
Female age, years					
At consent	32 (30-34)	33 (30-36)	0.035	32 (30-34)	0.147
At oocyte retrieval	31 (29-34)	32 (29-35)	0.043	31 (29-34)	0.344
BMI, kg/m^2	22.7 ± 3.1	22.5 ± 3.1	0.545	22.5 ± 2.9	0.948
Previous live birth	68 (15.8)	13 (8.6)	0.029	8 (5.3)	0.258
Previous abortion	126 (29.2)	51 (33.8)	0.297	37 (24.5)	0.076
Duration of infertility, years	4 (2-6)	5 (3-7)	0.011	5 (3-6)	0.452
Indications for IVF			0.974		0.873
Tubal factor	172 (39.9)	62 (41.1)		53 (35.1)	
Male factor	63 (14.6)	21 (13.9)		25 (16.6)	
Ovulatory dysfunction	34 (7.9)	12 (7.9)		10 (6.6)	
Endometriosis	11 (2.6)	2 (1.3)		3 (2.0)	
Combined factors	134 (31.1)	48 (31.8)		52 (34.4)	
Unexplained	17 (3.9)	6 (4.0)		8 (5.3)	
Basal follicle-stimulating hormone, mIU/ml	6.5 ± 2.0	7.0 ± 2.4	0.026	6.9 ± 2.2	0.705
Previous oocyte retrievals	2 (1-2)	2 (1-3)	0.005	2 (1-3)	0.396
Previous failed embryo transfer cycles	3 (2-3)	2 (2-3)	<0.001	2 (2-3)	0.134
Previous embryos transferred	4 (3-5)	4 (3-5)	0.249	4 (3-5)	0.111
Previous good-quality embryos transferred	4 (3-5)	4 (3-4)	0.515	3 (3-4)	0.175
Fertilization method, No. (%)			0.190		0.635
IVF	288 (66.8)	92 (60.9)		96 (63.6)	
ICSI	143 (33.2)	59 (39.1)		55 (36.4)	
Original-trial prednisone allocation, No. (%)	207 (48.0)	75 (49.7)	0.728	77 (51.0)	0.818
**FET cycle characteristics**					
Endometrial thickness, mm	9.0 ± 1.5	9.2 ± 1.7	0.167	9.0 ± 1.5	0.218
Stage of embryo(s), No. (%)			<0.001		<0.001
D3		151 (100.0)			
D5	294 (68.2)			108 (71.5)	
D6	137 (31.8)			43 (28.5)	
**Pregnancy outcomes, No. (%)**					
Biochemical pregnancy	258 (59.9)	69 (45.7)	0.003	91 (60.3)	0.011
Clinical pregnancy	218 (50.6)	65 (43.0)	0.111	78 (51.7)	0.134
Pregnancy loss	78/258 (30.2)	11/69 (15.9)	0.018	27/91 (29.7)	0.043
Biochemical pregnancy loss	40/258 (15.5)	4/69 (5.8)	0.036	13/91 (14.3)	0.084
Clinical pregnancy loss	38/218 (17.4)	7/65 (10.8)	0.197	14/78 (17.9)	0.227
Live birth	180 (41.8)	58 (38.4)	0.471	64 (42.4)	0.482

Abbreviations: SBT, single blastocyst-stage embryo transfer; DET, double cleavage-stage embryo transfer; BMI, body mass index; No, number; IVF, *in vitro* fertilization, ICSI, intracytoplasmic sperm injection. Propensity scores were estimated using logistic regression based on age at oocyte retrieval, previous live birth,

duration of infertility, basal follicle-stimulating hormone, previous oocyte retrievals, previous failed embryo transfer cycles, and randomized prednisone/placebo allocation in the original trial.

Biochemical pregnancy loss was calculated among biochemical pregnancies and defined as failure to progress to clinical pregnancy. Clinical pregnancy loss was calculated among clinical pregnancies and defined as failure to result in live birth.

Supplementary Table S2. Pregnancy outcomes between SBT and DET among participants randomized to placebo in the original trial

**Pregnancy outcomes, No. (%)**	SBT (n=224)	DET (n=76)	*P*-value
Biochemical pregnancy	131 (58.5)	29 (38.2)	0.002
Clinical pregnancy	114 (50.9)	29 (38.2)	0.055
Pregnancy loss	35/131 (26.7)	2/29 (6.9)	0.022
Live birth	96 (42.9)	27 (35.5)	0.262

Abbreviations: SBT, single blastocyst transfer; DET, double cleavage-stage embryo transfer; No, number.

Supplementary Table S3. Perinatal Outcomes

	SBT	DET	*P*-value
**Number of live birth cycles**	180	58	
Gestational diabetes	32 (17.8)	7 (12.1)	0.307
Premature rupture of membrane	12 (6.7)	7 (12.1)	0.187
Gestational hypertension	8 (4.4)	3 (5.2)	0.732
Preeclampsia	6 (3.3)	0	0.340
Preterm delivery	13 (7.2)	10 (17.2)	0.025
Singleton	11/177 (6.2)	2/43 (4.7)	1.000
Twin	2/3 (66.7)	8/15 (53.3)	1.000
**Number of live newborns**	183	73	
Birthweight, g	3359.2 ± 636.8	2975.7 ± 593.9	<0.001
Singleton, mean ± SD [No.]	3390.2 ± 621.9 [177]	3338.6 ± 372.5 [43]	0.603
Twin, mean ± SD [No.]	2444.2 ± 334.2 [6]	2455.5 ± 446.8 [30]	0.954
Congenital anomalies	10 (5.5)	4 (5.5)	0.996

Abbreviations: SBT, single blastocyst transfer; DET, double cleavage-stage embryo transfer. SD, standard deviation; No, number.

Supplementary Table S4. Pregnancy outcomes among Day 5 SBT, Day 6 SBT, and DET cycles

**Pregnancy outcomes, No. (%)**	D5 SBT (n=294)	D6 SBT (n=137)	DET (n=151)	*P*-value
Biochemical pregnancy	194 (66.0)	64 (46.7)	69 (45.7)	<0.001
Clinical pregnancy	164 (55.8)	54 (39.4)	65 (43.0)	0.022
Pregnancy loss	58/194 (29.9)	20/64 (31.3)	11/69 (15.9)	0.059
Biochemical pregnancy loss	30/194 (15.5)	10/64 (15.6)	4/69 (5.8)	0.110
Clinical pregnancy loss	28/164 (17.1)	10/54 (18.5)	7/65 (10.8)	0.422
Live birth	136 (46.3)	44 (32.1)	58 (38.4)	0.016

Abbreviations: SBT, single blastocyst transfer; DET, double cleavage-stage embryo transfer; No, number.

Supplementary Table S5. Univariate and Multivariate Analysis with regard to Pregnancy Outcomes among Day 5 SBT, Day 6 SBT, and DET cycles

Outcome	D5 vs. D6 Adjusted OR (95% CI)	*P*-value	D3 vs. D5 Adjusted OR (95% CI)	*P*-value	D3 vs. D6 Adjusted OR (95% CI)	*P*-value
Biochemical pregnancy	2.39 (1.56 to 3.68)	<0.001	0.46 (0.30 to 0.70)	<0.001	1.09 (0.67 to 1.79)	0.720
Clinical pregnancy	2.02 (1.32 to 3.11)	0.001	0.62 (0.41 to 0.94)	0.024	1.24 (0.75 to 2.04)	0.405
Pregnancy loss	0.85 (0.44 to 1.63)	0.615	0.44 (0.21 to 0.95)	0.037	0.37 (0.15 to 0.91)	0.030
Biochemical pregnancy loss	0.98 (0.43 to 2.25)	0.966	0.42 (0.14 to 1.30)	0.133	0.40 (0.11 to 1.43)	0.158
Clinical pregnancy loss	0.80 (0.34 to 1.89)	0.612	0.48 (0.19 to 1.24)	0.130	0.39 (0.12 to 1.18)	0.096
Live birth	1.91 (1.22 to 3.00)	0.005	0.78 (0.51 to 1.20)	0.263	1.49 (0.88 to 2.51)	0.137

Abbreviations: SBT, single blastocyst transfer; DET, double cleavage-stage embryo transfer. OR, odds ratio; CI, confidence interval.

Data were adjusted for age at oocyte retrieval, previous live birth, duration of infertility, basal follicle-stimulating hormone, previous oocyte retrievals, previous failed embryo transfer cycles, randomized prednisone/placebo allocation in the original trial. For each pairwise comparison, the second group listed in the column heading was used as the reference group.

Supplementary Table S6. Stratified analysis

Age at oocyte retrieval	<35			≥35		
	SBT (n=363)	DET (n=106)	*P*-value	SBT (n=68)	DET (n=45)	*P*-value
Biochemical pregnancy	222 (61.2)	56 (52.8)	0.125	36 (52.9)	13 (28.9)	0.012
Clinical pregnancy	193 (53.2)	53 (50.0)	0.566	25 (36.8)	12 (26.7)	0.263
Pregnancy loss	61/222 (27.5)	9/56 (16.1)	0.079	17/36 (47.2)	2/13 (15.4)	0.043
Live birth	161 (44.4)	47 (44.3)	0.998	19 (27.9)	11 (24.4)	0.680

Previous retrieval(s)	=1			≥2		
	SBT (n=191)	DET (n=52)	*P*-value	SBT (n=240)	DET (n=99)	*P*-value
Biochemical pregnancy	110 (57.6)	27 (51.9)	0.465	148 (61.7)	42 (42.4)	0.001
Clinical pregnancy	92 (48.2)	25 (48.1)	0.991	126 (52.5)	40 (40.4)	0.043
Pregnancy loss	31/110 (28.2)	4/27 (14.8)	0.154	47/148 (31.8)	7/42 (16.7)	0.056
Live birth	79 (41.4)	23 (44.2)	0.710	101 (42.1)	35 (35.4)	0.250

Previous transfer(s)	=2			≥3		
	SBT (n=178)	DET (n=92)	*P*-value	SBT (n=253)	DET (n=59)	*P*-value
Biochemical pregnancy	105 (59.0)	44 (47.8)	0.080	153 (60.5)	25 (42.4)	0.011
Clinical pregnancy	92 (51.7)	41 (44.6)	0.267	126 (49.8)	24 (40.7)	0.207
Pregnancy loss	28/105 (26.7)	6/44 (13.6)	0.084	50/153 (32.7)	5/25 (20.0)	0.203
Live birth	77 (43.3)	38 (41.3)	0.758	103 (40.7)	20 (33.9)	0.335

Abbreviations: SBT, single blastocyst transfer; DET, double cleavage-stage embryo transfer.
